# Association between the bi-dimensional aspect of orthorexia and healthy behaviors among lebanese adolescents

**DOI:** 10.1186/s12888-022-04374-4

**Published:** 2022-11-21

**Authors:** Serena Samaha, Reine Azzi, Rana Rizk, Abir Sarray El Dine, Diana Malaeb, Souheil Hallit, Sahar Obeid, Michel Soufia

**Affiliations:** 1 School of Medicine and Medical Sciences, Holy Spirit University of Kaslik, P.O. Box 446, Jounieh, Lebanon; 2 Department of Natural Sciences, School of Arts and Sciences, Lebanese American University, Byblos, Lebanon; 3 Department of Biomedical Sciences, School of arts and Sciences, Lebanese International University, Beirut, Lebanon; 4 School of Pharmacy, Lebanese International University, Beirut, Lebanon; 5 College of Pharmacy, Gulf Medical University, Ajman, United Arab Emirates; 6 Applied Science Research Center, Applied Science Private University, 11931 Amman, Jordan; 7 Research Department, Psychiatric Hospital of the Cross, Jal Eddib, Lebanon; 8 Social and Education Sciences Department, School of Arts and Sciences, Lebanese American University, Jbeil, Lebanon

**Keywords:** Orthorexia Nervosa, Healthy Orthorexia, Healthy behaviors, Cigarette smoking, Waterpipe smoking, Alcohol, Physical activity, Adolescents, Lebanon

## Abstract

**Background:**

Recently, there has been a blooming focus on “eating healthy and clean”, with the ideal of being healthy becoming a popular lifestyle trend. Previous research suggested the presence of two forms of orthorexia: Orthorexia Nervosa (OrNe) and Healthy Orthorexia (HeOr). Taking into consideration that orthorexia thoughts are led by the desire to reach an optimal physical health, the dedication to a healthy living may require healthy lifestyle habits: smoking cessation, moderate alcohol intake, and increased physical activity. The main study aim was to determine, among Lebanese adolescents, the association between healthy behaviors and the two likely forms of orthorexia (OrNe and HeOr), given that the adolescent phase is a risky period in the development of eating disorders.

**Methods:**

A cross-sectional study, conducted between January and April 2022, enrolled 444 adolescents aged between 14 and 18 years, with an equitable random sample from all the Lebanese governorates (mean age 16.23 ± 1.15 years; 60.1% females). The Teruel Orthorexia Scale (TOS) was used to assess orthorexic eating tendencies. The Lebanese Waterpipe Dependence Scale, the Fagerström Test for Nicotine Dependence and the Alcohol Use Disorder Identification Test scales, all validated in adolescents, were used to evaluate the association between orthorexia and healthy behaviors.

**Results:**

Higher TOS OrNe scores were significantly and highly correlated with the TOS HeOr scores (*r* = 0.68; *p* < 0.001). In the bivariate analysis, waterpipe smoking was weakly but significantly associated with more OrNe, whereas more physical activity was significantly and moderately associated with more HeOr. After adjusting over all sociodemographic characteristics, other behaviors and the other dimension of orthorexia, more alcohol use disorder was significantly but strongly associated with higher OrNe, whereas more physical activity remained significantly associated with higher HeOr.

**Conclusion:**

In light of our results, OrNe and HeOr might be considered as different entities, mainly in their associations with alcohol consumption and physical activity. This supports the hypothesis of OrNe being a new form of eating disorder, whereas HeOr possibly showing protective characteristics. Orthorexia is still a topic of controversy, especially in the probable presence of two facets that are still difficult to differentiate.

## Background

There has been a blooming focus on “eating healthy and clean”, mainly in the past two decades. Besides the concept of skinniness and thinness, the ideal of being healthy has become a popular lifestyle trend with a leading role [1]. Research has revealed that a healthy diet is linked, in a positive way, to a longer life span and a reduced probability of developing chronic disease [2]. However, there is a fine line between healthy eating and pathological healthy eating [3].

The desire to eat healthy food is not in itself a disorder [4, 5]. However, as mentioned by Donini et al., when it turns into an obsessive painstaking habit with the absence of balance, it could subsequently lead to Orthorexia Nervosa (OrNe) [4, 5].

The term Orthorexia Nervosa comes from the combination of the two Greek words *orthos* (= right) and *orexis* (= appetite) [6]. They portray a “maniacal obsession for healthy food” [5]. Orthorexia Nervosa, conceived in 1997 by Steven Bratman [1], is characterized by a harsh attention on healthy food consumption and a pathological obsession with its biological purity. Moreover, due to lack of sufficient studies on the topic, orthorexia is not yet identified as a disease in either the Diagnostic and Statistical Manual of Mental Disorders, 5th Edition (DSM-5) published in 2013 [7] or in the International Classification of Diseases (ICD-11) approved in 2019 [8].

Barrada & Roncero suggested, in addition to the pathological form- Orthorexia Nervosa (OrNe), the presence of a non-pathological concern in clean and healthy eating, known as Healthy Orthorexia (HeOr) [9]; thus, unveiling a bi-dimensional facet of orthorexia. Key elements of HeOr are a “healthy interest with diet, healthy behavior with regard to diet, and eating healthily as part of one’s identity” [10]. The latest reliable tool for the evaluation of orthorexia, the Teruel Orthorexia Scale (TOS), constructed by Barrada & Roncero in 2018, was recently validated in its Arabic version, in Lebanon [11, 12]. The TOS covered the two distinguishable sides of orthorexia: OrNe and HeOr. On one hand, HeOr includes individuals interested in a healthy nutritional diet. On the other hand, OrNe determines the unfavorable emotional and social burden of completing a stiff eating plan [9].

HeOr, the non-pathological aspect of orthorexia, could be associated with healthy routine behaviors. However, OrNe is described as a “health fanatic” eating obsession, given that all their thoughts and actions are driven by the desire to reach ideal physical health and purity [5]. To achieve this goal, the physical training must take part into this healthy lifestyle to be fully completed. Exercising helps individuals on a psychological level by being a method to control stress, refresh, and enhance positive affect [13]. Orthorexia has been positively linked in multiple previous studies to an increased level of physical activity, making it a component that might turn up into a possible sport addiction. Previous authors have found that high levels of involvement in sports predicted a greater orthorexic risk in Hungarian university students and Polish adolescents, respectively [14, 15]. Furthermore, Rudolph et al. (2018) stated that exercise addiction, training hours per week, were also linked to a greater orthorexic risk in the context of German fitness sports [16]. However, other authors did not find any correlation [17–19].

Healthy behaviors include as well smoking cessation (cigarettes and waterpipe) and moderate alcohol intake. It may sound logical that non-users of alcohol and nicotine derivatives would have a higher tendency for orthorexia; and inversely, people with HeOr do not use alcohol and nicotine due to their interest in maintaining a healthy lifestyle. On one hand, previous authors found that OrNe, alcohol consumption and tobacco use were unconnected in adults [14, 18] and in adolescents [20]. On the other hand, Hyrnik et al. detected a link between non-smokers and OrNe in adolescents [15]. Roncero et al. showed an absence of relation between orthorexia and smoking, but concluded that non-alcohol drinkers would exhibit orthorexia more than frequent alcohol drinkers [19]. Harmful use of alcohol consumption and smoking have been major health concerns worldwide. The consumption of alcohol has been increasing among adolescents [21], especially in Lebanon, where an increase of 48% in adolescents’ drunkenness was witnessed between 2005 and 2011 [22]. Furthermore, Lebanon ranks first regarding smoking prevalence in the Middle-East [23]. For instance, waterpipe use has an expending popularity in Lebanon, with 35% of the adolescents, between 13 and 15 years old, having previously tried it due to its appealing different flavors and huge popularity [24]. In Lebanon, the waterpipe use prevalence is worryingly high and triples that of current cigarette smoking mainly in youth [25]. A new Lebanese study showed that 24.81% of Lebanese adolescents smoked waterpipe and that 69.5% had high waterpipe dependence (scores of ≥ 10) [26]. Recently, OrNe was positively associated with the consumption of unhealthy substances (higher alcohol use, cigarette and waterpipe smoking), while HeOr was negatively correlated with these mentioned behaviors, in Lebanese adults [27].

The association between demographic variables and orthorexia is still ambiguous. Multiple studies showed that women have higher symptoms of orthorexia compared with men [6, 12, 19, 28–30]; whereas others found the exact opposite [5, 31, 32] or even no difference at all between the two genders [15, 17, 33–36]. These discordant results may be because these studies were conducted in different countries with cultural dissimilarities and with different assessment tools of Orthorexia.

Studies on the effect of the socioeconomic status (SES) are mixed. Some studies showed either the presence or the absence of relationship between the SES and orthorexic eating traits [32, 37, 38]. The SES and educational levels were suggested to be correlated with orthorexia, indicating that a higher level is predictive of a higher orthorexia level [39, 40] and successively associated with healthier eating and purchases [41]. In Lebanon, higher SES was associated with higher nutritional knowledge than lower SES in Lebanese youth [42]; however, due to the economic collapse, the adherence to a healthy lifestyle was likely decreased [11].

The biggest part of the literature has shed light on the pathological form of this problematic eating behavior and its presence among adults. Although, it is commonly known that the adolescent phase is a risky period in the constitution and appearance of eating disorders (ED) [15], the effect of orthorexia on adolescents has received significantly less attention than in adults. During the state of puberty, individuals aspire building their self-esteem and struggle in finding balance between a proper diet and an optimal body weight [43]. This is especially the case in Lebanon, where adolescents are going through a hard time with the economic collapse and the unstable political situation, as well as the COVID-19 pandemic. This triple crisis left most of Lebanese adolescents struggling causing more mental health issues, unhealthy behaviors adaptation, and lower adherence to a healthy lifestyle. Only two international studies recruited samples of adolescents [15, 43]. With 41% of the sample formed of adolescent students, Lucka et al. (2019) concluded that subjects going to junior secondary school had the highest risk of orthorexia and those attending senior secondary school had the lowest risk [43]. Until now, available studies do not offer conclusive findings concerning the correlation between Orthorexia, and substance use and physical activity. No significant relationships or negative relationships between Orthorexia and the mentioned variables were found in most studies. This can be explained by the fact that previous research was done using scales of poor psychometric properties and questionable validity. Plus, the evaluation of orthorexia in adolescents is scarce. Therefore, the main purpose of the study is to determine the correlation between lifestyle behaviors (cigarette/waterpipe smoking, alcohol drinking, and physical activity) and the two probable aspects of orthorexia (OrNe and HeOr) among Lebanese adolescents, with the sociodemographic factors as confounding variables. In this study, we hypothesize that HeOr would be associated with less smoking and alcohol dependence and more physical activity; while OrNe would be associated with the opposite findings.

## Methods

### Study design

This cross-sectional study, conducted between January and April 2022, enrolled 444 adolescents aged between 14 and 18 years, using an equitable random sample from all Lebanese governorates. A snowball sampling technique, using social media platforms, was carried out during this period due to the COVID-19 pandemic outbreak in Lebanon as a result of the implemented social distancing. For this reason, to have an easy way for proceeding with the data collection, a soft copy of the questionnaire was conceived using the Google forms platform. The objectives and general requirements were passed on online to each adolescent before participating in this conducted research. No credit was received for participation. The inclusion criterion was any Lebanese adolescent aged between 14 and 18 years; while the exclusion criterion was any Lebanese adolescent who refused to participate in the current study.

### Minimal sample size calculation

According to the G-power software [44], taking an alpha error of 5%, a power of 80%, an effect size f2 = 2%, and taking into account 10 factors to be embarked on the multivariable analysis, the minimal sample size recommended was 395 adolescents.

### Measures & materials

Originally, the questionnaire was developed in Arabic. The sociodemographic information of each adolescent was assessed in the beginning of the built questionnaire (age, gender, residency governorate). The Household Crowding Index (HCI) was calculated by dividing the number of persons living in the same house by the number of rooms; the bathrooms and the kitchen were excluded. The HCI reflects the SES of the family; thus, the higher the HCI, the lower the SES [45]. The physical activity index was calculated by multiplying the duration, frequency and intensity of the physical activity Weary-Smith KA. Validation of the physical activity index (PAI) as a measure of total activity load and total kilocalorie expenditure during submaximal treadmill walking. University of Pittsburgh; 2007. The second part collected information about cigarette and waterpipe smoking and alcohol drinking with the following scales:

#### Teruel Orthorexia Scale (TOS)

The Teruel Orthorexia Scale (TOS) was used to assess orthorexia [9]. The definitive version, with its 17 items, covered the differentiable, yet linked aspects of orthorexia: OrNe and HeOr, which are assessed, respectively, with 8 and 9 items [9]. The responses are arranged on a 4-point Likert scale ranging from 0 (Completely disagree) to 3 (Completely agree) [9]. The Arabic version of TOS has been validated as a predictable tool to evaluate the presence of orthorexic tendencies in Lebanese adolescents in 2021 [11]. The original Spanish study revealed an adequate internal consistency with a Cronbach’s alpha value of 0.85 for TOS HeOr and 0.81 for TOS OrNe [9]; while in the validated Arabic version, they were 0.829 for TOS HeOr and 0.853 for TOS OrNe [11]. In our study, Cronbach’s alpha values were 0.91 for TOS HeOr and 0.90 for TOS OrNe.

#### Lebanon Waterpipe Dependence Scale-11 (LWDS-11)

The Lebanon Waterpipe Dependence Scale-11 (LWDS-11) [46] is a test composed of 11 items providing the clearest and most interpretable solution for the assessment of the waterpipe dependence. It has been validated among Lebanese adolescents with a Cronbach’s alpha value of 0.96 [26]. The original study had a Cronbach’s alpha equal to 0.83 [46]. Higher scores indicate higher waterpipe addiction. In the current study, the Cronbach’s alpha value for this scale was 0.79.

#### Fagerström Test for Nicotine Dependence (FTND)

The Fagerström Test for Nicotine Dependence (FTND), validated among Lebanese adults with a Cronbach’s alpha of 0.780 [47], consists of a 6-item scale measuring nicotine dependence with a possible range from 0 to 10. It is used to evaluate the intensity of physical addiction to nicotine due to cigarette smoking. The FTND assesses the load of cigarette consumption, the drive to use, and the dependence/addiction. It has been validated among Lebanese adolescents with a Cronbach’s alpha of 0.75 [48]. The higher the score, the more intense the physical dependence on nicotine [49]. In this study, the Cronbach’s alpha value for this scale was 0.74.

#### Alcohol Use Disorders Identification Test (AUDIT)

The Alcohol Use Disorders Identification Test (AUDIT) is a self-reported screening tool for hazardous alcohol consumption [50]. It consists of a 10-items core questionnaire assessing the alcohol consumption, drinking attitudes, and drinking consequences [51]. It has been validated among Lebanese adolescents with a Cronbach’s alpha of 0.978 [20]. One study found that the AUDIT scale has a good sensitivity and specificity for the identification of alcohol disorders among adolescents aged between 13 and 19 years [52]. In this study, the Cronbach’s alpha value for this scale was 0.92.

### Statistical analysis

Data analysis was conducted using the 23rd version of the SPSS software [53]. The normality of distribution of the age, physical activity index, TOS OrNe, TOS HeOr, FTND, and LWDS scores were confirmed via a calculation of the skewness and kurtosis; values for asymmetry and kurtosis between − 2 and + 2 are considered acceptable to prove normal univariate distribution [54]. The household crowding index and AUDIT score were converted into log10 values; the latter showed normal distribution and were therefore used in the rest of the analysis. The assumption of normality is reinforced, with these conditions, in samples more than 300 [55]. Pearson correlations were assessed between TOS OrNe, TOS HeOr scores, and other variables (cigarette/waterpipe smoking, alcohol use, gender, age, household crowding index (HCI), and physical activity). The partial correlations were evaluated between the different factors and the variables while controlling for the other aspect of orthorexia. In addition, partial correlations were chosen as regression models for two reasons. First, Pearson and partial correlation can be compared in an easier way, as the two of them align between − 1 to + 1. Second, regression coefficients and partial correlations both generate the exact inferential results because they have equal p-values. In psychological analysis, correlations of 0.1, 0.2, and 0.3 were considered as having a small, medium, and large effect sizes, respectively [56]. Significance is considered with a p-value < 0.05.

## Results

A total of 444 adolescents, aged between 14 and 18 years, were enrolled in this study (mean age 16.23 ± 1.15 years; 60.1% females). The results showed that 76 (17.1%) of the adolescents smoked cigarettes, 100 (22.5%) smoked waterpipe, and 65 (14.6%) drank alcohol. The mean scores for the continuous variables were as follows: household crowding index (1.28 ± 0.87), physical activity index (28.46 ± 20.74), TOS OrNe (14.32 ± 5.96), and TOS HeOr (20.46 ± 6.84). Among smokers, the mean FTND score was 3.50 ± 2.85 and 14.04 ± 6.42 for the LWDS, whereas the mean AUDIT score was 7.60 ± 7.97 among alcohol drinkers.

### Association between the orthorexia scales (OrNe and HeOr), lifestyle habits and sociodemographic variables

Higher TOS OrNe scores were significantly and highly correlated with the TOS HeOr scores (*r* = 0.68; *p* < 0.001). In the bivariate analysis, waterpipe smoking was weakly but significantly associated with more OrNe, whereas more physical activity was significantly and moderately associated with more HeOr. After adjusting over all sociodemographic characteristics (age, sex, household crowding index and physical activity), other behaviors and the other dimension of orthorexia, the results showed that more alcohol use disorder was significantly but strongly associated with higher OrNe, whereas more physical activity remained significantly associated with higher HeOr (Table 1).

**Table 1:** Zero order and partial correlations between variables (*N*=444)

	Cigarette smoking	Waterpipe smoking	Alcohol drinking	Age	Gender	HCI	PA
**TOS OrNe**							
Zero order	*r*= 0.06	***r*= 0.13**	*r*= 0.17	*r*= 0.05	*r*= 0.06	*r*= 0.02	*r*= 0.09
Partial	*r*= 0.06	*r*= -0.20	***r*= 0.37**	*r*= -0.14	*r*= 0.08	*r*= 0.17	*r*= -0.03
**TOS HeOr**							
Zero order	*r*= -0.05	*r*= 0.03	*r*= -0.22	*r*= 0.01	*r*= 0.09	*r*= -0.07	***r*= 0.22**
Partial	*r*= -0.10	*r*= 0.23	*r*= -0.46	*r*= 0.20	*r*= -0.09	*r*= -0.11	***r*= 0.14**

*HCI* Household crowding index, *PA* Physical activity, *TOS* Teruel Orthorexia Scale, *OrNe* Orthorexia nervosa, *HeOr* Healthy orthorexia. Values in bold correspond to statistically significant correlations (*p*<0.05). Zero order corresponds to correlations between variables without any adjustment over other variables. Partial corresponds to correlations adjusted for age, gender, household crowding index, physical activity index and the other orthorexia dimension

## Discussion

### Orthorexia nervosa vs. healthy orthorexia

Our study’s results revealed that the two dimensions of orthorexia (HeOr and OrNe) were significantly and highly correlated to each other with the used constructs. Past studies showed that OrNe is positively correlated with psychopathology, while HeOr is oppositely related to psychopathology [9, 57]. A recent study showed OrNe and HeOr as being distinctive forms, with HeOr representing a non-pathological aspect [58]. Moreover, the motives of food choices for the two aspects are relatively distinct [10]. After all, these two forms may not be different and might evaluate the same aspect of orthorexia. Moreover, the two forms are closely related to each other than to the health-related behaviors (cigarette/waterpipe smoking, alcohol drinking, and physical activity). Additional research is required to define the similarities and dissimilarities between OrNe and HeOr as measured by the TOS, even if the biggest part of the literature considers them as basically similar.

### Waterpipe and cigarette smoking

As it is known, nicotine is a stimulant substance; yet, it has some relaxing effects. It has an impact on performance, mood and attitude and it decreases hunger [46]. Therefore, smoking can be used as a coping mechanism [59]. In the beginning, we hypothesized that HeOr would be associated with less cigarette/waterpipe smoking and OrNe would be associated with more nicotine use. However, in our bivariate analysis, waterpipe smoking was weakly but significantly associated with more OrNe, while cigarette smoking was not associated with HeOr nor OrNe.

Waterpipe has exceeded cigarettes in its popularity among Lebanese youth (up to 35% of teenagers) and the Middle Eastern countries [24, 60, 61], even with its complex preparation process. Many elements led to the spread of wastepipe’s popularity: harm misconceptions and easy accessibility. Due to its different fruity flavors, waterpipe gained a smoother image in people’s mind, making it more popular and appealing to adolescents [62]. Moreover, researchers found that waterpipe is seen as an ‘‘aesthetic enjoyable experience’’ [63, 64]. This might be also due to waterpipe smoking being more socially accepted than cigarette smoking among adolescents, with a particular characteristic of being shared with a family member. The concept of the “positive” attributes of waterpipe, like relaxing and socializing, led to the increase and encouragement of its consumption [65]. Awad et al. detected a significant relationship between waterpipe smoking and social media use [66].

However, we argue that the association between OrNe or HeOr with cigarette is irrelevant in our study. Varga et al., 2014 and Oberle et al., 2019 pointed to an absence of significance between nicotine usage and orthorexia [14, 67]. Other studies also found no significant correlation between orthorexia and smoking status [18, 38, 68–70]. Those results were incoherent with the finding of Hyrnik et al., 2016 [15]: the presence of a connection between orthorexia and non-smokers. This is explained by the fact that cigarette smoking is not considered as a healthy behavior, with HeOr perceived as a securing behavior [57]. OrNe was associated with higher levels of tobacco consumption, while there was no association between these factors and HeOr in a recent study by Zickgraf & Barrada [58]. However, the exact role of smoking is not yet known in terms of risk factor for the development of orthorexia, needing extra research for a better comprehension.

### Alcohol use

It is logical that the obsession with a healthy lifestyle would involve the avoidance alcohol and that non-alcohol drinkers would have greater levels of OrNe. Previous studies found that alcohol use was not related to OrNe [14, 18, 38]. In Hyrnik’s study, alcohol was the most common abusive substance consumed by Polish adolescents [15]. We assumed that Orthorexia, in its two aspects (HeOr and OrNe) would be correlated in an opposite matter with alcohol consumption in order to be healthy: less alcohol in HeOr and more alcohol in OrNe. However, in our study, we found that the AUDIT score was not associated with TOS OrNe, but turned to be significantly but strongly associated with higher OrNe when controlling over the other sociodemographic variables and HeOr. Harmful alcohol consumption has been a major health concern in the world as its consumption has been increasing among adolescents [21]. A reason behind this finding might be due to the absence of law enforcement in Lebanon, making the purchase of alcoholic drinks easier with no respect to age limitations [22, 71]. Alcohol use among adolescents might be due to social media influence mixed with their curiosity, feeling of power or ability to deal with their stress [72, 73]. Moreover, being a substrate of the GABAergic receptors, alcohol helps in decreasing stress and anxiety; this explains why individuals with an insecure attachment, specifically anxious attachment [74], may have greater alcohol consumption. Despite the obsessions and compulsions with healthy eating, Zickgraf & Barrada detected that OrNe was associated with more unhealthy eating and other lifestyle behaviors (substance use and sedentary lifestyle), making OrNe and HeOr distinct constructs [58]. Also, in our results, the TOS HeOr scores were not significantly associated with problematic alcohol use before and after variables adjustment. This could be explained by assuming that OrNe is a novel aspect of eating disorder, while HeOr could be perceived as a securing behavior [57]. Yet, the results lead us to question whether the constructs used were appropriate to separate OrNe and HeOr with future research needed to clarify the conflicting results.

### Physical activity

“A fit body represents health and is a symbol of good living; an obese body is regarded as lazy, emotionally weak, and unattractive” [75]. This goes well with our study’s results: higher physical activity is significantly and moderately related to more HeOr after adjustments made over the other variables. Our result confirmed a part of our hypothesis: HeOr being correlated with more physical activity. People use exercise as an approach to reduce the social physique anxiety they have through creating an appealing body form [76]. HeOr is seen to be directly related to the aim of maintaining a healthy lifestyle; even though a Lebanese study found that adults with high HeOr tendency have healthier life behaviors, except for physical activity [27]. Moreover, an analysis of the workout motivation revealed that individuals exercise to improve their psychological health and not only to reach an optimal physical health, but also to deal with stress, revitalize, and increase positive affect [13]. Hence, the physical activity effect on physical and mental health are outstanding [77].

However, in our study, as shown elsewhere, no correlation between OrNe and physical activity was found [17–19]. Previous authors detected a high level of physical activity being associated with greater OrNe risk [14, 15]. A recent study done by Brytek-Matera et al. revealed that young Polish and Italian adults with high orthorexia levels showed high-intensity physical activity in comparison with young adults having low Orthorexia levels [77]. Thus, from a desire to reach the ideal health status, high level Orthorexic individuals may enroll in high-intensity physical activity in the interest of its health benefits [77]. All in all, the relation between OrNe and physical activity seems to be tricky. Thus, the difference between OrNe and HeOr can be explained by the motive behind their behaviors: for OrNe, weight control is the motive, whereas for HeOr, health content is the motive. These findings raise questions on the precise role played by exercise in orthorexia, and on whether exercise habits should be viewed as a symptom of orthorexia [14].

### Age

No significant relationship between age and orthorexia was detected, in line with previous studies [38, 78, 79]. Yet, Lucka et al. found the highest orthorexia risk was seen in adolescents aged between 13 and 16 years old and the lowest risk was in the age group of 16 and 19 years old [43]. This vulnerable age group being more prone to OrNe behaviors mainly because they are busy building their self-image; therefore, struggling in finding a perfect balance between a proper diet and a prime body weight yet [43]. In addition, our finding was different compared with other studies [5, 34], stating that orthorexia increases with increased age and might be a related factor to healthy eating behavior [80].

### Gender

Our study results did not show any correlation between the two forms of orthorexia and gender, which is in line with previous studies [15, 17, 35, 36, 68, 79, 81]. No gender differences were detected also in Turkish performance artists [38], and resident medical doctors [34]. Therefore, these results do not approve the popular the fact that more awareness and a better perception of nutrition is seen in women [82]. Previous studies showed the positive correlation between orthorexia and women [6, 12, 19, 28–30, 39, 83]. In fact, females feel pressured to reach a perfect body shape matching with society’s norms [84] and start pushing themselves to build this stereotyped thin body. Specifically, in the Lebanese society, the image of body is judged as being a “female problem”, while males focus more on physical appearance and muscles’ perception instead of healthy eating [85]. The disparity of the gender constitution in our sample must be taken into consideration (60.1% of females). Future research with approximately equal gender distribution sample are needed to clarify the association.

### Clinical implications

Orthorexia is still a topic of controversy. More research targeting Orthorexia is needed due to the inconsistency in the obtained results and the multiple methods used for its assessment in previously conducted studies. The main purpose of our study was to determine the correlation between lifestyle behaviors (cigarette/waterpipe smoking, alcohol drinking, and physical activity) and the two probable aspects of Orthorexia among Lebanese youth. In light of our results, OrNe and HeOr might be distinct in their correlation with waterpipe smoking, alcohol consumption, and physical activity. Overall, since Orthorexia is not yet defined as a psychiatric disorder, our findings on the correlates of Orthorexia can help professionals in detecting them easily in high-risk individuals, for a better understanding and exploration of Orthorexia. The relationship between Orthorexia and exercise should be additionally explored as a risk factor for the development of Orthorexia, and followed up regularly for any sign of sport addiction. Moreover, concerning lifestyle habits, particularly alcohol consumption and cigarette/waterpipe smoking, enforcing a minimum legal age for alcoholic drinks and tobacco purchase and consumption is needed. Adolescents in Lebanon are prone to these unhealthy behaviors due to low alcohol prices and availability, wide marketing on the media and social acceptance of waterpipe smoking. The findings of the current study can help professionals look cautiously for these unhealthy behaviors in suspected individuals with Orthorexia. Finally, our results will help in building a greater perspective about Orthorexia in Lebanese adolescents and guiding future research for a better understanding of this phenomenon to be able to asses and diagnose Orthorexia in an easier way. Awareness in this vulnerable age group should be taken seriously, especially at schools, on the matter of eating disorders EDs and unhealthy substance use. Preventive distinction between the pathological (OrNe) and the non-pathological (HeOr) orthorexic behaviors in adolescence is of highly importance for a better understanding of this emerging topic.

### Limitations

This research has few limitations. Conclusions cannot be drawn concerning the causality because of the cross-sectional design of the study with temporality issues. The screening of orthorexia was made through an online questionnaire instead of a clinical diagnostic interview. Thus, the accuracy of the responses could not be confirmed and more assessment is needed by psychological experts to support the obtained results. Female students were more represented in the study (60.1%), possibly restricting the generalizability of the results found. Furthermore, the majority of adolescents recruited were school students. The snowball technique was used as a recruitment strategy due to the COVID-19 pandemic; even though representativeness and implication of the results to the Lebanese population are not ensured. Although significant, the correlation coefficients between OrNe and HeOr are small; therefore, the interpretation of the results should be done cautiously. Not all co-variables were explored in this paper, adding a residual confounding bias to our study. Consequently, this work paves the way for future studies to address other variables such as gastrointestinal disorders and health anxiety that could impact Orthorexia’s features. Eating disorders were not measured or discussed although adolescents have a high incidence of eating disorders symptomatology.

## Conclusion

Presently, in light of our results, OrNe and HeOr might be considered as different entities, mainly in their correlation with waterpipe smoking, alcohol consumption, and physical activity, where OrNe was associated with unhealthier behaviors, while HeOr was associated with a healthy lifestyle, mainly more physical activity. This supports the hypothesis of OrNe being a new form of eating disorder, whereas HeOr possibly showing protective characteristics. The results lead us to question whether the constructs used were appropriate to separate OrNe and HeOr. Until now, all conducted studies do not provide compatible results concerning the correlation between orthorexia and lifestyle habits: substance use (smoking and alcohol drinking) and physical activity. Orthorexia is still a topic of controversy, especially in the probable presence of two facets: HeOr and OrNe, which are still difficult to separate. Longitudinal and prospective studies are essential in providing more knowledge about this bi-dimensional feature of orthorexia: healthy vs. pathological, understanding better the correlates of orthorexia behaviors and investigating the effect of other co-variables (such as gastrointestinal disorders and health anxiety).

## Data Availability

All data generated or analyzed are not publicly accessible in order to protect the privacy of the participants’ identities. The dataset supporting the conclusions is available from the corresponding author upon appropriate request.

## References

[1] Bratman S. Health Food Junkie. Yoga Journal (September/October) http://www.orthorexia.com/original-orthorexia-essay; 1997.

[2] Katz DL, Meller S (2014). Can we say what diet is best for health?. Annu Rev Public Health.

[3] Missbach B, Barthels F (2017). Orthorexia Nervosa: moving forward in the field. Eat Weight Disord.

[4] Lorenzo M, Donini, Donini LM, Marsili D, Graziani MP, Imbriale M, et al. BRIEF REPORT e28 Orthorexia nervosa: Validation of a diagnosis questionnaire. 2005;10: 28. 10.1007/BF03327537 16682853

[5] Donini LM, Marsili D, Graziani MP, Imbriale M, Cannella C (2004). Orthorexia nervosa: A preliminary study with a proposal for diagnosis and an attempt to measure the dimension of the phenomenon. Eat Weight Disord.

[6] Haddad C, Obeid SS, Akel M, Honein K, Akiki M, Azar J (2019). Correlates of orthorexia nervosa among a representative sample of the Lebanese population. Eat Weight Disord..

[7] American Psychiatric Association (2013). Diagnostic and statistical manual of mental disorders: DSM-5. Fifth edition ed.

[8] World Health Organization. International classification of diseases for mortality and morbidity statistics. 11th revision. 2018. [Internet]. Available from: https://icd.who.int/browse11/l-m/en [cited 16 Dec 2019).

[9] Barrada JR, Roncero M (2018). Bidimensional Structure of the Orthorexia: Development and Initial Validation of a New Instrument. Anales de psicología (Murcia, Spain)..

[10] Depa J, Barrada JR, Roncero M (2019). Are the Motives for Food Choices Different in Orthorexia Nervosa and Healthy Orthorexia?. Nutrients..

[11] Mhanna M, Azzi R, Hallit SS, Obeid S, Soufia MM (2022). Validation of the Arabic version of the Teruel Orthorexia Scale (TOS) among Lebanese adolescents. Eat Weight Disord..

[12] Awad E, Obeid SS, Sacre H, Salameh PP, Strahler J, Hallit SS. Association between impulsivity and orthorexia nervosa: any moderating role of maladaptive personality traits? Eat Weight Disord. 2021;1. 10.1007/s40519-021-01186-5 33840074

[13] Oberle CD, Watkins RS, Burkot AJ (2018). Orthorexic eating behaviors related to exercise addiction and internal motivations in a sample of university students. Eat Weight Disord.

[14] Varga M, Thege BK, Dukay-Szabó S, Túry F, van Furth EF (2014). When eating healthy is not healthy: orthorexia nervosa and its measurement with the ORTO-15 in Hungary. BMC Psychiatry.

[15] Hyrnik J, Janas-Kozik M, Stochel M, Jelonek I, Siwiec A, Rybakowski JK (2016). The assessment of orthorexia nervosa among 1899 Polish adolescents using the ORTO-15 questionnaire. Int J psychiatry Clin Pract.

[16] Rudolph S (2018). The connection between exercise addiction and orthorexia nervosa in German fitness sports. Eat weight disorders.

[17] Dunn TM, Bratman S (2016). On orthorexia nervosa: A review of the literature and proposed diagnostic criteria. Eat behaviors: Int J.

[18] Strahler J, Hermann A, Walter B, Stark R (2018). Orthorexia nervosa: A behavioral complex or a psychological condition?. J Behav addictions.

[19] Roncero M, Barrada JR, Perpiñá C (2017). Measuring Orthorexia Nervosa: Psychometric Limitations of the ORTO-15. Span J Psychol.

[20] Hallit J, Salameh P, Haddad C, Sacre H, Soufia M, Akel M (2020). Validation of the AUDIT scale and factors associated with alcohol use disorder in adolescents: results of a National Lebanese Study. BMC Pediatr..

[21] Snyder LB, Milici FF, Slater M, Sun H, Strizhakova Y (2006). Effects of Alcohol Advertising Exposure on Drinking Among Youth. Arch Pediatr Adolesc Med..

[22] Ghandour L, Afifi R, Fares S, El Salibi N, Rady A (2015). Time Trends and Policy Gaps: The Case of Alcohol Misuse Among Adolescents in Lebanon. Subst Use Misuse..

[23] Salti N, Brouwer E, Verguet S (1982). The health, financial and distributional consequences of increases in the tobacco excise tax among smokers in Lebanon. Soc Sci Med.

[24] Bahelah R, DiFranza JR, Ward KD, Eissenberg T, Fouad FM, Taleb ZB (2017). Waterpipe smoking patterns and symptoms of nicotine dependence: The Waterpipe Dependence in Lebanese Youth Study. Addict Behav.

[25] Jawad M, Nakkash RT, Mahfoud Z, Bteddini D, Haddad P, Afifi RA (2015). Parental smoking and exposure to environmental tobacco smoke are associated with waterpipe smoking among youth: results from a national survey in Lebanon. Public health (London).

[26] Hallit S, Obeid S, Sacre H, Salameh P (2021). Lebanese Waterpipe Dependence Scale validation in a sample of Lebanese adolescents. BMC Public Health.

[27] Hallit S, Barrada JR, Salameh P, Sacre H, Roncero M, Obeid S (2021). The relation of orthorexia with lifestyle habits: Arabic versions of the Eating Habits Questionnaire and the Dusseldorf Orthorexia Scale. J Eat disorders.

[28] Parra-Fernandez ML, Rodríguez-Cano T, Onieva-Zafra MD, Perez-Haro MJ, Casero-Alonso V, Muñoz Camargo JC (2018). Adaptation and validation of the Spanish version of the ORTO-15 questionnaire for the diagnosis of orthorexia nervosa. PLoS ONE.

[29] Dell’Osso L, Abelli M, Carpita B, Pini S, Castellini G, Carmassi C (2016). Historical evolution of the concept of anorexia nervosa and relationships with orthorexia nervosa, autism, and obsessive-compulsive spectrum. Neuropsychiatr Dis Treat.

[30] Sanlier N, Yassibas E, Bilici S, Sahin G, Celik B (2016). Does the rise in eating disorders lead to increasing risk of orthorexia nervosa? Correlations with gender, education, and body mass index. Ecol food Nutr.

[31] Malmborg J, Bremander A, Olsson MC, Bergman S (2017). Health status, physical activity, and orthorexia nervosa: A comparison between exercise science students and business students. Appetite.

[32] Fidan T, Ertekin V, Işikay S, Kırpınar I (2010). Prevalence of orthorexia among medical students in Erzurum, Turkey. Compr Psychiatr.

[33] Brytek-Matera A, Fonte ML, Poggiogalle E, Donini LM, Cena H (2017). Orthorexia nervosa: relationship with obsessive-compulsive symptoms, disordered eating patterns and body uneasiness among Italian university students. Eat Weight Disord.

[34] Bagci Bosi AT, Camur D, Guler C (2007). Prevalence of orthorexia nervosa in resident medical doctors in the faculty of medicine (Ankara, Turkey). Appetite.

[35] Oberle CD, Samaghabadi RO, Hughes EM (2017). Orthorexia nervosa: Assessment and correlates with gender, BMI, and personality. Appetite.

[36] Farchakh Y, Hallit S, Soufia M. Association between orthorexia nervosa, eating attitudes and anxiety among medical students in Lebanese universities: results of a cross-sectional study. Eat Weight Disord. 2019;24:683. 10.1007/s40519-019-00724-6. 10.1007/s40519-019-00724-6 31183627

[37] Missbach B, Hinterbuchinger B, Dreiseitl V, Zellhofer S, Kurz C, König J. When Eating Right, Is Measured Wrong! A Validation and Critical Examination of the ORTO-15 Questionnaire in German. PLoS One. 2015;10:e0135772. 10.1371/journal.pone.0135772 10.1371/journal.pone.0135772 PMC4539204 26280449

[38] Aksoydan E, Camci N. Prevalence of orthorexia nervosa among Turkish performance artists. Eat Weight Disorders. 2009;14:33–7. 10.1007/BF03327792 10.1007/BF03327792 19367138

[39] Strahler J, Haddad C, Salameh P, Sacre H, Obeid S, Hallit S. Cross-cultural differences in orthorexic eating behaviors: Associations with personality traits. Nutrition (Burbank, Los Angeles County, Calif). 2020;77:110811. 10.1016/j.nut.2020.110811 10.1016/j.nut.2020.110811 32447071

[40] Varga M, Dukay-Szabó S, Túry F, van Furth Eric F (2013). Evidence and gaps in the literature on orthorexia nervosa. Eat Weight Disord.

[41] Pechey R, Jebb SA, Kelly MP, Almiron-Roig E, Conde S, Nakamura R (2013). Socioeconomic differences in purchases of more vs. less healthy foods and beverages: Analysis of over 25,000 British households in 2010. Soc Sci Med (1982).

[42] Nabhani-Zeidan M, Naja F, Nasreddine L (2011). Dietary Intake and Nutrition-Related Knowledge in a Sample of Lebanese Adolescents of Contrasting Socioeconomic Status. FoodNutr Bull.

[43] Łucka I, Janikowska-Hołoweńko D, Domarecki P, Plenikowska-Ślusarz T, Domarecka M (2019). Orthorexia nervosa - a separate clinical entity, a part of eating disorder spectrum or another manifestation of obsessive-compulsive disorder?. Psychiatr Pol.

[44] Faul F, Erdfelder E, Lang AG, Buchner A. G* Power 3: A flexible statistical power analysis program for the social, behavioral, and biomedical sciences. Behav Res Methods. 2007 May;39(2):175–91. 10.3758/bf03193146 17695343

[45] Melki IS, Beydoun HA, Khogali M, Tamim H, Yunis KA (2004). Household crowding index: a correlate of socioeconomic status and inter-pregnancy spacing in an urban setting. J Epidemiol Commun Health..

[46] Salameh P, Waked M, Aoun Z (2008). Waterpipe smoking: Construction and validation of the Lebanon Waterpipe Dependence Scale (LWDS-11). Nicotine Tob Res.

[47] Salameh P, Khayat G, Waked M (2013). The Lebanese Cigarette Dependence (LCD) Score: a Comprehensive Tool for Cigarette Dependence Assessment. Int J Behav Med.

[48] Nonnemaker JM, Homsi G (2006). Measurement properties of the Fagerström Test for nicotine dependence adapted for use in an adolescent sample. Addict Behav.

[49] Carpenter MJ, Baker NL, Gray KM, Upadhyaya HP (2010). Assessment of nicotine dependence among adolescent and young adult smokers: A comparison of measures. Addict Behav.

[50] Saunders JB, Aasland OG, Babor TF, De La Fuente JR, Grant M. Development of the Alcohol Use Disorders Identification Test (AUDIT): WHO Collaborative Project on Early Detection of Persons with Harmful Alcohol Consumption-II. Addiction (Abingdon, England). 1993;88:791–804. 10.1111/j.1360-0443.1993.tb02093.x 8329970

[51] Bohn MJ, Babor TF, Kranzler HR (1995). The Alcohol Use Disorders Identification Test (AUDIT): validation of a screening instrument for use in medical settings. J Stud Alcohol.

[52] Knight JR, Sherritt L, Harris K, Gates EC, Chang G. Validity of Brief Alcohol Screening Tests Among Adolescents: A Comparison of the AUDIT, POSIT, CAGE, and CRAFFT. doi: 10.1097/01.ALC.0000046598.59317.3A. 10.1097/01.ALC.0000046598.59317.3A 12544008

[53] IBM Corp (2015). Released 2015. IBM SPSS Statistics for Windows, Version 23.0.

[54] George D. SPSS for Windows Step by Step: A Simple Study Guide and Reference, 17.0 update, 10th edition, Pearson Education India, 2011.

[55] Mishra P, Pandey C, Singh U, Gupta A, Sahu C, Keshri A (2019). Descriptive statistics and normality tests for statistical data. Ann Card Anaesth.

[56] Ozer DJ, Funder DC (2020). Corrigendum: Evaluating Effect Size in Psychological Research: Sense and Nonsense. Advanc Methods Pract Psychol Sci..

[57] Barthels F, Barrada JR, Roncero M (2019). Orthorexia nervosa and healthy orthorexia as new eating styles. PLoS ONE.

[58] Zickgraf  HF, Barrada JR (2022). Orthorexia nervosa vs. healthy orthorexia: relationships with disordered eating, eating behavior, and healthy lifestyle choices. Eat Weight Disord Stud Anorexia Bulimia Obes.

[59] Wise MH, Weierbach F, Cao Y, Phillips K (2017). Tobacco use and attachment style in Appalachia. Issues Mental Health Nurs..

[60] World Health Organization. Advisory note: waterpipe tobacco smoking. Health effects, research needs and recommended actions for regulators (2nd edition). 2015. Retrieved from http://www.who.int/tobacco/publications/prod_regulation/waterpipesecondedition/en/. Accessed 25 Sept 2022.

[61] Maziak W, Taleb ZB, Bahelah R, Islam F, Jaber R, Auf R (2014). The global epidemiology of waterpipe smoking. Tob Control..

[62] Eissenberg T, Ward PhD, Smith-Simone KD,PhD, Maziak SPhD, Wasim MD. Ph.D. Waterpipe Tobacco Smoking on a U.S. College Campus: Prevalence and Correlates. J Adolesc Health. 2008;42:526–9. 10.1016/j.jadohealth.2007.10.004. 10.1016/j.jadohealth.2007.10.004 PMC2362063 18407049

[63] Maziak W, Ward KD, Eissenberg T (2004). Factors related to frequency of narghile (waterpipe) use: the first insights on tobacco dependence in narghile users. Drug Alcohol Depend.

[64] Ward KD, Eissenberg T, Rastam S, Asfar T, Mzayek F, Fouad MF (2006). The tobacco epidemic in Syria. Tob Control.

[65] Smith-Simone S, Maziak W, Ward K, Eissenberg  T (2008). Waterpipe tobacco smoking: Knowledge, attitudes, beliefs, and behavior in two U.S. samples. Nicotine Tobacco Res.

[66] Awad E, Hallit R, Haddad C, Akel M, Obeid S, Hallit S. Are social media use disorders associated with higher addictions (alcohol, smoking and waterpipe) among Lebanese adults?. 2020. doi: 10.21203/rs.3.rs-15654/v1. 10.1111/ppc.12963 34716597

[67] Oberle CD, Klare DL, Patyk KC (2019). Health beliefs, behaviors, and symptoms associated with orthorexia nervosa. Eat Weight Disord.

[68] Almeida C, Vieira Borba V, Santos L (2018). Orthorexia nervosa in a sample of Portuguese fitness participants. Eat Weight Disord.

[69] Oberle CD, De Nadai AS, Madrid AL (2021). Orthorexia Nervosa Inventory (ONI): development and validation of a new measure of orthorexic symptomatology. Eat weight disorders.

[70] Parra-Fernandez M-, Rodriguez-Cano T, Onieva-Zafra M-, Perez-Haro MJ, Oberle CD, Fernandez-Martinez E, et al. Prevalence of orthorexia nervosa in university students and its relationship with psychopathological aspects of eating behavior disorders. BMC Psychiatry. 2018;18: 1–8. doi: 10.1186/ s12888-018-1943-0. 10.1186/s12888-018-1943-0 PMC6234674 30424750

[71] Nasser Yassin R, Afifi N, Singh R, Saad L Ghandour. “There Is Zero Regulation on the Selling of Alcohol”: The Voice of the Youth on the Context and Determinants of Alcohol Drinking in Lebanon. 2018. 10.1177/1049732317750563 29307267

[72] Coleman LM, Cater SM (2005). A Qualitative Study of the Relationship Between Alcohol Consumption and Risky Sex in Adolescents. Arch Sex Behav.

[73] Massad SG, Shaheen M, Karam R, Brown R, Glick P, Linnemay S (2016). Substance use among Palestinian youth in the West Bank, Palestine: a qualitative investigation. BMC Public Health..

[74] Molnar DS, Sadava SW, DeCourville NH, Perrier CPK (2010). Attachment, Motivations, and Alcohol: Testing a Dual-Path Model of High-Risk Drinking and Adverse Consequences in Transitional Clinical and Student Samples. Can J Behav Sci.

[75] Crawford R. Cultural influences on prevention and the emergence of a new health consciousness. In: Anonymous Taking Care. Cambridge University Press; 1987. pp. 95–114.

[76] Hausenblas HA, Brewer BW, Van Raalte JL (2004). Self-Presentation and Exercise. J Appl sport Psychol.

[77] Brytek-Matera A, Pardini S, Szubert J, Novara C. Orthorexia Nervosa and Disordered Eating Attitudes, Self-Esteem and Physical Activity among Young Adults. Nutrients. 2022 Mar;18(6):1289. 14(. 10.3390/nu14061289 PMC8948728 35334945

[78] Bundros J, Clifford D, Silliman K, Neyman Morris M (2016). Prevalence of orthorexia nervosa among college students based on Bratman’s test and associated tendencies. Appetite.

[79] Grammatikopoulou MG, Gkiouras K, Markaki A, Theodoridis X, Tsakiri V, Mavridis P (2018). Food addiction, orthorexia, and food-related stress among dietetics students. Eat Weight Disord..

[80] Shelton NJ (2005). What not to eat: inequalities in healthy eating behaviour, evidence from the 1998 cottish Health Survey. J Public Health.

[81] Barnes MA, Caltabiano ML (2016). The interrelationship between orthorexia nervosa, perfectionism, body image and attachment style. Eat Weight Disord.

[82] Kiefer I, Kunze M, Kiefer I, Rathmanner T, Kunze M. Review Keywords Gender Differences Nutritional behaviour Weight reduction Body perception Eating and dieting differences in men and women.

[83] Hallit S, Brytek-Matera A, Obeid S (2021). Orthorexia nervosa and disordered eating attitudes among Lebanese adults: Assessing psychometric proprieties of the ORTO-R in a population-based sample. PLoS ONE.

[84] Borchert J, Heinberg L (1996). Gender Schema and Gender Role Discrepancy as Correlates of Body Image. J Psychol.

[85] Moustafa S, Tawbe Z, Sleiman F, Daouk SE, Koubar M, Hoteit M (2017). Body Image Perception in Association with Healthy Lifestyle Behaviour’s in Lebanese Men and Women. Int J School Cogn Psychol..

